# Recent Developments on *Salmonella* and *Listeria monocytogenes* Detection Technologies: A Focus on Electrochemical Biosensing Technologies

**DOI:** 10.3390/foods14234139

**Published:** 2025-12-02

**Authors:** Keletso Eunice Ipeleng, Usisipho Feleni, Valentine Saasa

**Affiliations:** 1 Department of Life and Consumer Sciences, College of Agriculture and Environmental Sciences, Florida Campus, University of South Africa (UNISA), Roodepoort 1709, South Africa; 66131626@mylife.unisa.ac.za; 2 Institute for Nanotechnology and Water Sustainability, College of Science, Engineering and Technology, University of South Africa (UNISA), Johannesburg 1710, South Africa; felenu@unisa.ac.za

**Keywords:** foodborne diseases, food safety, *Salmonella* species, *Listeria monocytogenes*, biosensors, electrochemical biosensors

## Abstract

Foodborne pathogens such as *Salmonella* species and *Listeria monocytogenes* are leading causes of foodborne illness outbreaks worldwide, posing significant public health and economic challenges. For years, culture-based methods and culture-independent methods have been widely used for pathogen detection; however, their limitations have become increasingly apparent, i.e., longer turnaround times, and they have lower specificity and selectivity. Recent innovations in molecular, immunological, spectroscopic, and biosensing technologies offer promising alternatives for rapid, sensitive, and on-site detection of these pathogens. In this review paper, we provide an overview of the conventional and emerging detection technologies for *Salmonella* species and *Listeria monocytogenes* in food matrices, and their limitations. Emphasis is placed on electrochemical biosensors for *L. mono* and *Salmonella* detection and their integration in food testing and monitoring. Finally, we conclude and discuss the future perspectives of electrochemical biosensors.

## 1. Introduction

Bacterial foodborne illnesses pose a significant health threat, particularly in developing countries. The World Health Organization estimates that foodborne illnesses affect approximately 600 million people annually, resulting in over 420,000 deaths, with the majority occurring in low- and middle-income countries. Among the most common and high-risk foodborne pathogens are *Salmonella* spp. and *Listeria monocytogenes* (*L. mono*). The Centers for Disease Control (CDC) has estimated that 1600 people catch listeriosis annually, resulting in approximately 260 deaths [1]. The risk and severity of listeriosis are notably elevated among susceptible groups, including immunocompromised individuals, pregnant women, the elderly, and infants, with an associated 16–25% fatality rate despite treatment [2]. Additionally, its ability to grow at refrigeration temperatures raises significant food safety concerns [3].

**Figure 1:**
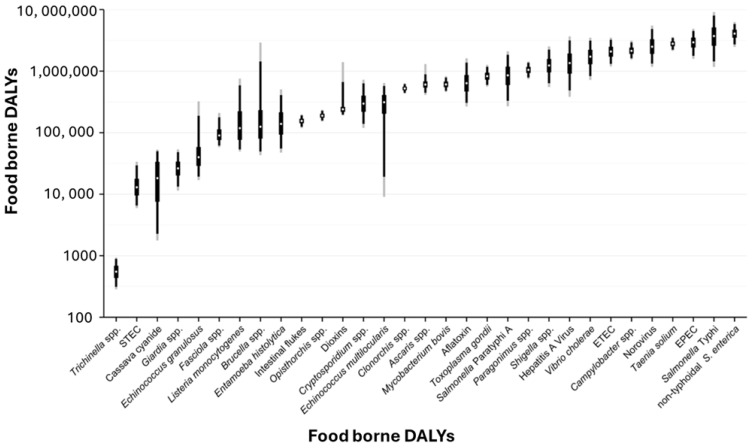
Global ranking of hazards, including bacteria, responsible for foodborne illnesses, expressed as disability-adjusted life years (DALYs) [6].

*Salmonella* species, on the other hand, a causative agent of salmonellosis, has been reported to be the second leading pathogen that causes foodborne illnesses, with the highest percentage of hospitalisations after campylobacteriosis [2,4]. Each year, *Salmonella* causes 93.8 million cases of gastroenteritis and approximately 155,000 deaths globally. *Salmonella* consists of over 2500 diverse typhoidal and nontyphoidal serotypes (NTS), with non-typhoidal *Salmonella* serotypes being reported as the major cause of diarrheal diseases worldwide [5]. Again, the population groups most affected by salmonellosis are individuals with compromised immune systems, pregnant women, and the elderly [2]. Figure 1 shows a trend in studies conducted for the detection of pathogens in various food matrices. Figure 1 illustrates the median rates of foodborne disability-adjusted life years (DALYs) per 100,000 population caused by pathogenic bacteria [6].

To date, various *Salmonella* and *L. mono* detection techniques exist with different principles and detection mechanisms [7,8,9,10,11,12,13], as shown in Table 1.

Currently, culture-based methods have demonstrated a high success rate and offer reliable and standardized procedures for the detection of pathogens [14,15,16]. However, the nutrient agar media used have low sensitivity, which implies increased risk of foodborne disease outbreaks as the risk goes unnoticed, and target pathogens cannot be distinguished from other microorganisms in low-selective media [17]. In addition, culture-based methods are time-intensive as they involve multi-step processes to achieve definitive identification of the microorganism. These steps include media preparation, pre/initial enrichment preparation, selective and differential plating, sample incubations, and a series of confirmatory tests and strain typing [18,19]. The entire culture process, from preliminary isolation to final species confirmation, typically takes between 2 and 5 days. This is too long for food manufacturers and suppliers, as they require swift testing to monitor their food products for the presence of pathogens to ensure their products meet legislative standards for pathogen levels in certain food categories, thereby preventing the distribution of unsafe food products. Also, border inspection agencies require rapid test results to ensure unsafe food products are not being imported [18].

Furthermore, the food matrices’ heterogeneity, uneven distribution, and often low microbial levels in food samples, as well as the presence of non-target microbes, may compete and interfere with target microbes, leading to misinterpretation of test results. Additionally, culture-based methods have shown a high limit of detection (LOD) for microorganisms that are in an injured or a viable but nonculturable (VBNC) state [18]. Lastly, Selim and colleagues [19] have also reported that only about 1% of bacteria can be cultured in laboratory settings, as cultures often lack the necessary nutrients, growth factors, and optimal environmental conditions, such as pH and temperature. Other limitations include the inability to detect fastidious bacteria, potential for cross-contamination, bias toward dominant species, and limitation to functional studies [20].

Improved methods of analysis, such as molecular-based assays, polymerase chain reaction (PCR), have been developed to shorten the total assay time with high specificity. This method offers excellent bacterial detection with high affinity [8]. It is also rapid and sensitive. However, despite being sensitive, rapid, and specific, PCR involves amplification of DNA small segments through a repeating cycle of replication [21]. PCR cannot distinguish between DNA derived from live and dead cells, as it amplifies DNA from both live and dead cells, and this can lead to false-positive results [18,21,22]. In addition, due to the genetic similarities among various bacteria, common DNA may be amplified, increasing the risk for false-positive results by the detection of normal flora of the corneal external environment. Also, PCR is highly susceptible to contamination compared to culture and staining techniques because of its sensitivity to DNA [21]. Moreover, it has limitations for on-site testing and is costly, and requires specialized personnel [23,24].

Conversely, immunological-based assays such as ELISA and chemiluminescent enzyme immunoassay (CLEIA), rely on antibodies that recognize and attach to specific proteins or lipopolysaccharides found on the external membrane of the bacteria. Cross-reactivity of antibodies used with other bacterial species or subspecies of the targeted bacteria may lead to false-positive results. In addition, just like traditional culture-based detection methods, immunoassays involve a series of processing steps and desktop instrumentation [22], and although they are effective diagnostic tools, they can be impractical for on-site testing, are costly, and require highly skilled personnel [25].

**Figure 2:**
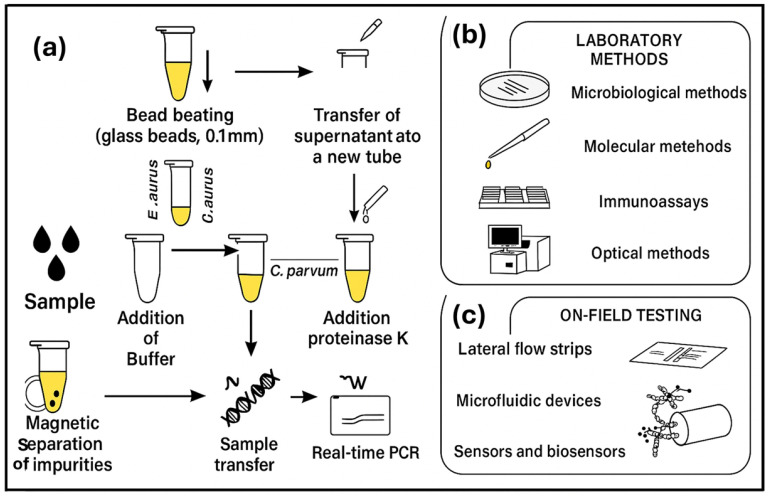
(**a**) Visual representations of the DNA extraction procedure, the first step in many molecular-based assays used for bacterial detection. (**b**) Laboratory techniques, namely, molecular methods, immunoassays, optical and separation techniques used to detect bacteria, and (**c**) on-field testing methods, namely, lateral flow strips, microfluidic devices, sensors, and biosensors [20].

Spectroscopy-based methods and mass spectrometry-based methods, while ideal for bacterial detection, face limitations in certain areas. These limitations include high cost, and results may depend on environmental conditions. Just like molecular and immunological-based methods, they also require sample preparation, skilled personnel, and expensive equipment, as shown in Figure 2 [9].

One way to improve food safety and monitoring is to employ rapid, accurate, cost-effective, real-time, specific, and sensitive detection methods or techniques, as well as providing on-site detection to achieve better results. Biosensing technology has been introduced as an alternative tool to meet these essential requirements. Among the various biosensor platforms, electrochemical biosensors have gained significant attention due to their high sensitivity, portability, low cost, ease of use, and miniaturization. These sensors employ biorecognition elements such as antibodies, nucleic acids, or bacteriophages immobilized on an electrode surface to detect target pathogens through measurable electrical signals [26,27]. This review focuses on recent advancements in the detection of *Salmonella* and *L. mono*, with particular emphasis on electrochemical biosensing technologies. We examine both conventional and emerging detection methods, highlight their respective limitations, and explore the role of electrochemical biosensors in enhancing food safety monitoring.

## 2. Overview of Foodborne Pathogens and Their Impact

Foodborne pathogens pose a major threat to public health and place a substantial economic burden on society, resulting in significant costs related to healthcare and lost productivity, with over 200 foodborne illnesses identified [28,29,30]. Common bacteria causing foodborne illnesses include *Salmonella*, *L. mono*, *Bacillus cereus*, *Staphylococcus aureus*, *Cronobacter sakazakii*, *Vibrio species*, *Clostridium perfringes*, *Clostridium botulinum*, *Yersinia enterocolitica*, *Campylobacter jejuni*, *Shigella* spp., *Escherichia coli*, Shiga toxin-producing *Escherichia coli* [3,29,30,31], with *Salmonella* and *Listeria* being reported as the leading causes of foodborne diseases, resulting in high numbers of reported death cases, among foodborne pathogens [2]. According to the United States (US) 2023 data analysis, the estimated annual cost of foodborne illnesses in the US was around $75 billion. Deaths made up about 56% of the average cost, while chronic health outcomes accounted for approximately 31%. The costliest pathogen was Nontyphoidal *Salmonella*, with estimated costs totalling around $17.1 billion [29]. Hoffmann et al., [28] also reported *Salmonella* as the costliest pathogen ($2.8 billion in chicken and 1.9 billion in pork) in his study, while *Listeria* was reported to cost approximately $4 billion primarily due to deaths and chronic outcomes from pregnancy-associated cases [29].

### 2.1. Listeria Monocytogenes

*Listeria* species has been reported as a prominent contributor to foodborne illnesses [32]. Among the genus *Listeria*, *L. mono* and *Listeria ivanovii* (*L. ivanovii*) are the only species considered pathogenic for humans [33]. *L. mono* is intracellular and Gram-positive [34]. It is psychotropic and ubiquitous in nature, i.e., widely dispersed in the natural environment, food processing environments, as well as within the gastrointestinal tract of healthy humans and animals. These features enable it to cause contamination in foods, especially in ready-to-eat food products such as mixed salads (both vegetables and fruits), burgers, sausage rolls, pizza, cold deli meats, desserts, seafood salad, and unpasteurised milk and dairy products such as soft cheeses [1,2,34,35]. It is reported that 2621 listeriosis cases were reported in 2019, with persons over the age of 65 years being affected most. *L. mono* has been confirmed as a serious zoonotic disease, with a 92% hospitalization rate and a 17.6% mortality rate [2].

For these reasons, and especially for the protection of the consumer and public health, rapid and accurate detection of *Listeria* species in food is essential to allow food manufacturers time to quarantine and/or recall affected food batches and perform risk assessment, control, and prevention promptly without hindering their food supply [36].

### 2.2. Salmonella Species

*Salmonella* infections, particularly non-typhoidal infections, contribute significantly to global morbidity and mortality [8,37]. Salmonellosis is caused by the consumption of food contaminated with *Salmonella*. In 2017, an estimated 14.3 million cases of typhoid and paratyphoid fever occurred worldwide, resulting in approximately 136,000 deaths. *Salmonella enterica* serotype Typhi is a primary cause of enteric fever [8]. *Salmonella* is a rod-shaped and Gram-negative bacterium that belongs to the *Enterobacteriaceae* family, with optimum growth at 37 °C [2]. It comprises over 2500 serotypes, and the most common serotypes associated with human illness are *Salmonella enterica* serovar *Typhimurium* (*S. Typhimurium*) and *S. enterica* serovar *Enteritidis* (*S. enteritidis*) [38,39]. Foods that are commonly identified as vehicles of salmonellosis to humans include animal products such as eggs, poultry, meat, and dairy products [5].

The common occurrence of salmonellosis has underscored the need for efficient and effective techniques for *Salmonella* spp. identification, detection, and monitoring at the earliest stage possible to prevent potential food safety issues.

## 3. Conventional Detection Methods of Foodborne Pathogens

Researchers have conducted numerous studies to compare existing foodborne pathogen detection and confirmation techniques based on different approaches and mechanisms of detection [10,11,12,13,15]. These methods can be categorized into culture-based methods [10,15], molecular-based methods [11], immunological-based methods [12], and spectroscopy-based assays [13].

Traditional culture-based methods are the oldest bacterial detection methods that mainly rely on microbial culturing on selective or general media for isolation of specific bacteria of interest [40,41]. Molecular-based methods have emerged as alternatives to culture-based techniques [18,42]. They encompass the analysis of microbial genomes to detect specific target(s) within the microbial genome and can be categorized into two major groups, namely, the protein/antigen targeting technique and the nucleic acid targeting technique, depending on the biological markers used [43,44]. Immunological-based assays rely on specific antibody–antigen binding interaction to quantitatively and qualitatively determine the microbial presence in samples through immunological reactions [38]. Lastly, spectroscopy techniques are widely used to study the interactions between matter and electromagnetic radiation [45].

### 3.1. Culture-Based Methods

Traditional culture-based methods are widely considered the “gold standard” for microbiological food analysis [18]. They involve culturing and plate counting using various types of nutrient agar media to promote the growth of target microorganisms or selectively isolate specific species [46,47]. Culture-based methods rely on the bacterial ability to proliferate and multiply on growth media, forming visible colonies. They remain the most preferred choice for many food testing laboratories due to their simplicity, affordability, and ability to provide both quantitative and qualitative information on the type and number of viable microorganisms present in the food samples [48].

Bradford and colleagues [15] conducted a study on the detection of *Salmonella* in poultry (chicken feed and chicken caecal) using both culture-based methods and culture-independent diagnostic tests (CIDTs), metabarcoding (16S), qPCR, and shotgun metagenomic sequencing, to compare the limit of detections (LODs) for *Salmonella* between these methods. The study was also intended to explore the impact of non-selective enrichment on LOD. The samples were spiked with known quantities of *Salmonella enteritidis*, and the method accurately detected *Salmonella.* Despite the success of the *Salmonella* detection, the process was time-consuming and labour-intensive as it involved a series of steps such as sample preparation, overnight *Salmonella* culture preparation, spiking procedure, growing bacteria in selective broths and agar, and enrichment broth dilution test. In addition, the sample matrix had a great effect on the outcome of the results, as *Salmonella* could be detected at much lower spike-in levels in feed samples, which have lower microbial abundance than in caecal contents, which have high microbial abundance.

Another study [14] investigated the shelf-life assessment of *L. mono* on an industrial RTE salad using conventional culture-based methods and molecular methods, namely, propidium monoazide, quantitative PCR, and genetic profiling, respectively. Samples were kept at various temperatures, 4 °C, 12 °C, and 16 °C, and the analysis was performed at day 0, 4, and 8. All samples were prepared according to ISO 6887-2:2003 [49]. The culture-based method for *L. mono* detection was carried out according to ISO 11290-1 [50], while *L. mono* enumeration was performed according to ISO 11290-2:2017 [51]. Acceptable results were only obtained for samples that were stored at 4 °C, considering the results that were obtained for hygiene indicators. A total of 30 *L. mono* presumptive colonies were obtained throughout the study [14], as shown in Table 2.

As shown in Table 2, thirty presumptive colonies were identified as *L. mono*. However, only ten (33.3%) colonies were confirmed as *L. mono* by PCR, which were assigned to serogroups IIa, IIb, and IVb. The authors have explained that this may be due to misinterpretation of the characteristic colonies, since *L. ivanovii* colonies may have the same morphological aspect as *L. mono*. This can lead to false-positive results. In addition, their study’s findings also demonstrated the ability of PMAxx-qPCR to obtain higher quantification (>3 log cfu/g) of *L. mono* compared to the culture-based quantification, which exhibited relatively low numbers (<1 log cfu/g) for all temperatures, suggesting the method’s inability to detect microbes that are in a viable but nonculturable state. This work has demonstrated that the culture-based methods alone are not sufficient for *L. mono* detection and confirmation in RTE foods shelf-life assessment studies. They need to be coupled with other rapid, specific, and sensitive methods, such as PCR, to mitigate the potential risk for consumers.

### 3.2. Culture-Independent Methods

Culture-independent methods (CIM) in microbiology refer to molecular approaches that are used to analyze microorganisms directly from samples without requiring cultivation in a laboratory first, enabling the study of microorganisms that might be difficult or impossible to grow using traditional culture-based methods. Common molecular techniques include PCR-based techniques [11,52,53], loop-mediated isothermal amplification (LAMP) [42], and next-generation sequencing (NGS) [54]. Different culture-independent methods were developed as an alternative approach to traditional culture-based methods for bacterial detection in food due to their rapidity, sensitivity, and specificity. PCR is a laboratory technique that uses a special enzyme, DNA polymerase, also known as Taq polymerase, to amplify short segments of DNA [55]. LAMP, on the other hand, is defined as an isothermal nucleic amplification technique that amplifies DNA at constant temperature using two or three primer sets along with a DNA polymerase [56]. Finally, NGS is defined as a technique that enables fast and simultaneous sequencing of millions of DNA segments, revealing detailed information about an organism’s genetic composition, variations, epigenetic modifications, and gene expression profiles [57].

The use of molecular-based methods showed success in accelerating testing methods and procedures compared to culture-based methods [58]. The application of PCR-based methods has revolutionized the detection of foodborne pathogens by enabling rapid, specific, and sensitive detection in food products [11]. Ndraha and colleagues [11] developed a rapid detection and identification method for the three primary implicated microbes behind foodborne outbreaks, namely, *L. mono*, *Salmonella*, and *Staphylococcus aureus* (*S.aureus*) in broth and chicken meats using a PCR array combined with an automated magnetic bead-based system and CE assay. The developed method demonstrated great success in detecting organisms of interest. These findings showed the model’s effectiveness in detecting pathogens at low concentrations in both samples. It is also important to note that these results were achieved without the need for sample enrichment, demonstrating the method’s ability to detect samples at relatively low levels, and that the whole process was completed in approximately 3.5 h. Table 3 presents the limit of detection obtained in tracking *S. enterica*, *L. mono*, and *S. aureus* in the broth and chicken meat samples.

Despite this achievement, the authors have also reported on the limitations of the developed model. Just like culture-based methods, their method is time-consuming and laborious due to multiple-step processes that need to be followed, some of which involve binding, washing, and elution. These steps contribute to extended handling time, rendering it less suitable for high-throughput scenarios. Furthermore, the column-based approach is most likely to lower DNA yield and purity, particularly when dealing with samples containing minimal DNA content or complex matrices [11].

On the other hand, LAMP has emerged as a powerful alternative to PCR for detecting bacteria in food products and clinical specimens [42,59]. It is defined as a molecular diagnostic tool that allows for rapid and exponential amplification of specific DNA or RNA targets at a set temperature, and unlike PCR, LAMP can produce a positive result in under 30 min and exhibits high sensitivity [60]. Fiore and team [41] applied a colorimetric LAMP assay to detect *L. mono* in cooked ham. They contaminated 37 ready-to-eat (RTE) meat samples with known *L. mono* concentrations and used a colorimetric LAMP assay to detect *L. mono*. Their analysis was performed in parallel with three other methods, namely, culture-based method, real-time PCR, and real-time LAMP PCR for results comparison and validation of the colorimetric LAMP assay. For real-time LAMP PCR and colorimetric LAMP, they used a set of six LAMP primers, including forward inner primer (FIP) and backward inner primer (BIP), two outer primers, F3 and B3, and two loop primers, LF and LB, that were targeting eight specific regions on the *L. mono hlyA* gene. The specificity and sensitivity of the real-time LAMP PCR and colorimetric LAMP were performed using non-target strains such as *E.coli*, *S.aureus*, *Pseudomonas aeruginosa*, *Citrobacter Freundel*, *Bacillus cereus*, *S. Typhimurium*, *S. enteritidis*, etc., including other *Listeria* species, *L. ivanovii*, *L.innocua*, and *L. seeligeri.* The authors obtained the same results, i.e., 25 positives and 12 negative test samples, using all four methods, showing the colorimetric LAMP assay’s ability to detect *L. mono* in food matrices. In addition, the colorimetric LAMP for *L. mono* detection, targeting the *hlyA* gene, compared to the traditional method, real-time PCR, and real-time LAMP PCR, demonstrated 100% specificity and sensitivity. However, despite the method being simple and cost-effective, it was not as rapid as results for colorimetric LAMP, real-time PCR, and real-time LAMP PCR only became available after 24 h, and the traditional method, long after 24 h. The food industry requires test results quickly for various reasons, including early product release and ensuring food safety. In addition, visual colour change tests are not always ideal due to various factors such as colour blindness, variety in visual interpretation, and subtle colour changes, which may lead to incorrect reporting of results.

**Figure 3:**
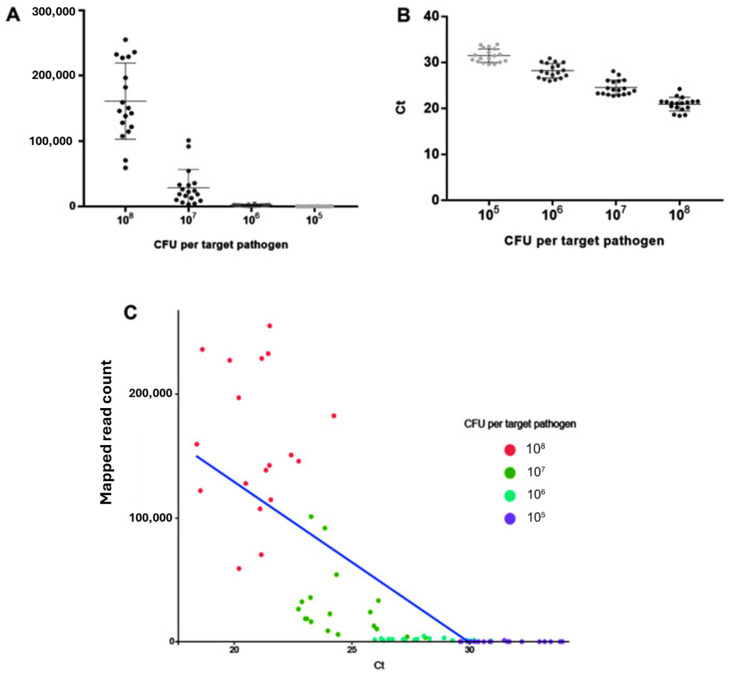
(**A**) NGS panel: Average of *Salmonella*, *L. mono*, and *E. coli* (10^5^–10^8^) gene reads, (**B**) qPCR: Ct values in a single replicate, and (**C**) average number of sequence reads that matched target pathogen genes and average Ct values [54].

Furthermore, another culture-independent molecular technique, the next-generation sequencing (NGS) panel, was developed and evaluated by Park and colleagues [54] in 2022. They developed NGS for simultaneous detection and identification of seven foodborne pathogenic bacteria, namely, *Salmonella enterica* serovar *Typhimurium*, *L. mono*, and five pathogenic strains of *E.coli* [(enteroinvasive *E.coli* (EIEC), enteropathogenic *E.coli* (EPEC), enterohemorrhagic *E.coli* (EHEC), enterotoxigenic *E.coli* (ETEC), and enteroaggregative *E.coli* (EAEC)], in six artificially contaminated fermented food products (three different types of yoghurt and three different types of Kimchi). Food samples were contaminated with 10^5^ to 10^8^ CFUs per target pathogen. One to three sets of primers were designed and evaluated to specifically target 13 specific virulence factor genes from the above-mentioned types of *E.coli*, *Salmonella enterica* serovar *Typhimurium*, and *L. mono*, respectively. Multiplex real-time PCR was used as a control to verify the NGS panel detection, while primer specificity to the genomic sequence of target pathogens was performed using cross-check and singleplex PCRs. DNA from the seven foodborne bacteria was used to prepare the library for NGS. Figure 3 shows results obtained for *E.coli*, *Salmonella*, and *L. mono* detection in fermented foods.

According to the results obtained, all 13 target genes were fully detected and identified in dilution factors 10^7^ to 10^8^ per pathogen in one NGS panel without any false positives, indicating the ability of the method to simultaneously detect multiple pathogens (*Salmonella*, *L. mono*, and pathogenic of *E. coli*) in one reaction. However, some difficulties were experienced with this method, as one false-positive result was obtained in a dilution factor of 10^6^ on the *fusA* gene of *L. mono* and many false-positive reads at 10^5^. In particular, the *fusA* gene of *L. mono* and the *stxA* gene of EHEC showed poor detection by the method. Additionally, when qPCR and NGS panel methods were compared, it was found that qPCR was more sensitive than the NGS panel method. Finally, the LOD and identification of the method were reported to be 10^7^ CFU.

### 3.3. Immunological-Based Assays

Immunological-based assays utilize antigen–antibody binding interactions for rapid and specific detection and identification. Enzyme-linked fluorescent assay (ELFA) [12], enzyme-linked immunosorbent assay (ELISA) [12,61], immunomagnetic separation [62], and lateral flow assay (LFA) [63] are among the most utilized immunoassays. ELISA and ELFA are widely accepted as the most preferred immunological-based techniques due to their cost-effectiveness, ease of use, and long-standing use [12].

Emine, 2023 [12] designed a study to determine the presence of *L. mono* and other *Listeria* species in retail RTE food products in Turkey, utilizing different methods, some of which include ELISA, ELFA, and PCR. Samples were contaminated with known different bacterial concentrations. For the detection by ELISA, sample pre-enrichment and enrichment steps were performed, as the domestic kit used requires enrichment prior to analysis. The ELISA analysis was carried out using the solution provided with the kit as per the manufacturer’s instructions. After incubation, the colour chart was used to visually evaluate the colour change in the wells, the results were recorded, and the absorbance was measured with the ELISA reader. The author completed this process in 100 min and was able to detect *L. mono* at a concentration of 1 cfu/mL. However, the method presents limited applicability due to the requirement for an enrichment process and costly equipment [12].

For the detection with ELFA, the procedure was carried out using a VIDAS immunoassay system (BioMérieux). Just like with the ELISA test, sample pre-enrichments and enrichments were performed prior to analysis. Samples together with controls were added to the wells of the strip. The strips were inserted into the VIDAS machine, and the test was carried out as per the manufacturer’s instructions. The SPSS 23.0 programme was used to analyze data obtained from the study. This process was completed in 90 min with a successful LOD of 1 cfu/mL [12]. However, just like the above-mentioned ELISA method, the ELFA method also has limited applicability due to sample enrichment processes.

Furthermore, Garrido-Maestu and team (2019) [62] developed a unique methodology for *L. mono* detection in smoked salmon samples. They combined immunomagnetic separation (IMS) with real-time recombinase polymerase amplification (qRPA) for fast and accurate detection of *L. mono.* They prepared different concentrations of *L. mono* containing samples, ranging from 2 to 9.3 × 10^2^ CFU/25 g. The detection technique was targeting the *hlyA* gene of *L. mono.* They analyzed 50 samples for four inoculation levels (0, 1–1.0 × 10, 1.0 × 10–1.0 × 10^2^, and 1.0 × 10^2^–1.0 × 10^3^ CFU/25 g). The methodology was run in parallel with the culture-based method for validation and multiplex qPC as a reference molecular method. The LoD50 was reported to be 6.3 CFU/25 g (95% CI2.6–14.1), and the RLOD, along with relative accuracy, specificity, and selectivity values higher than 90%. However, the analysis was completed in 24 h, which is too long for food manufacturers [62].

Gao et al. 2021 [63] developed an ultrasensitive LFA-based aptamer-magnetic separation and multifold gold nanoparticles for *S. Typhimurium* detection in milk samples. The method achieved LOD of 4.1 × 10^2^ CFU/mL. The authors used 13 microorganisms, including 3 *Salmonella* species, *S. Typhimurium*, *S. enteritidis*, and *S.dublin*, to evaluate the method’s specificity. No reaction with other non-target strains was observed, demonstrating high specificity. Despite the method’s ability to achieve relatively low LOD, the method is very complex, requires expensive equipment and reagents, and does not produce results immediately.

### 3.4. Spectroscopy-Based Methods

Spectroscopy techniques are a wide range of analytical techniques that analyze materials by examining their interaction with electromagnetic radiation [64] and have been used widely for bacterial detection [13,65]. Previously, molecular and immunological assays were widely used for *L. mono* and *Salmonella* detection in foods. Despite their rapidity, they have limitations, which led to the development of spectroscopic techniques. During the last years, spectroscopy-based methods such as mid-infrared (MIR) [13], Raman spectroscopy [65], and near-infrared spectroscopy (NIR) [63], have been used as complementary methods for bacterial detection due to their rapid “fingerprinting” and ability to provide molecular information [13].

Moreirinha and colleagues [13] have successfully used MIR spectroscopy to confirm the presence of *Salmonella* (*Salmonella enterica* sv *Nottingham*, Liverpool and Anatum serovars) and *Listeria* species (*L. mono*, *L. innocua*, and *L. ivanovii*) isolated from sausages, cheeses, and prepared dishes. The isolation procedure was performed according to ISO 6579 [66] and ISO 11290-1 [50], respectively. Instead of using biochemical tests to confirm presumptive colonies, the authors opted for MIR. The analysis of isolated bacterial strains was performed in an infrared spectrometer in a temperature-controlled room. The MRI enabled the authors to have a good assessment and discrimination of the analyzed *Salmonella* and *Listeria* species presumptive colonies down to their species or serogroups level, shortening the identification procedure by 2 days. Despite the methodology being deemed best and useful by authors, the confirmation process was carried out from the colonies grown in culture media.

Rodríguez-Lorenzo and team (2019) [65] have used a surface-enhanced Raman scattering (SERS) incorporated with a microfluidic chip for specific *L. mono* detection. They used *L. mono*-specific monoclonal antibody, Mab-C11E9, to functionalise the SERS-tagged gold nanoparticles. The resulting barcoded nanoparticles functionalized with the antibody were incubated in samples containing different *L. mono* concentrations as well as *Listeria innocua* and *S. Typhimurium* to determine the ability of the system to selectively detect target bacterium. The samples were then injected into a flow-focusing microfluidic device for the in-flow detection of the bacteria.

The system was able to detect *L. mono* down to 1 × 10^5^ CFU/mL. In addition, not only were the authors able to detect *L. mono* in a short period of time, but they were also able to distinguish *L. mono* from *Listeria innocua*, demonstrating high selectivity. In addition, the system demonstrated the ability to produce quick results as the overall preparation and system operation were conducted in 30 min, and data analysis, less than 2 min. Lastly, it is also important to note that this is the first time SERS tags are used for the in-flow detection of bacteria [65].

In 2018, Piara et al. [67] developed a method for fast detection of *Salmonella* in contaminated milk samples using near-infrared spectroscopy. Sample preparation includes a mixture of 5 mL milk sample and 1 mL of the *Salmonella* inoculum. From the solution, 1 mL was taken to the instrument and measured by NIR. The validation results indicate the method’s sensitivity and specificity, confirming that the samples were correctly identified. However, a near-infrared spectrometer showed sensitivity to fat and protein content in milk samples. In addition, the authors have also reported that analyzing liquid milk samples using NIR is challenging due to the wavelength range (different components absorb NIR radiation at specific wavelengths), the choice between reflectance or transmission, and sample preparation and presentation.

## 4. Biosensors for Pathogen Detection

**Figure 4:**
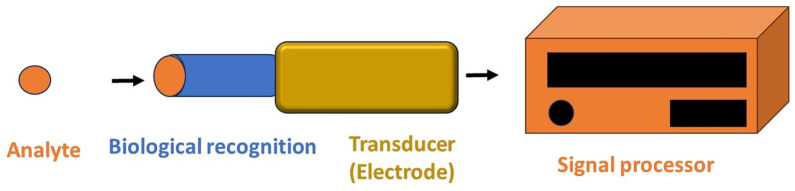
Basic design of a biosensor illustrating the major elements of a standard biosensor [72]. Adapted, with permission from Saasa et al., *Sensing and Biosensing*, Elsevier, 2025 [72].

Biosensors development has been fuelled by the need and desire for portable analytical tools that can produce accurate results in a short time and are suitable for on-site applications. Because of the advancements in nanotechnology, the development of biosensing technology has improved the speed and portability of analytical instrumentation. Thus, modern biosensor systems can deliver high-speed processing, high detection sensitivity and specificity, and quantitative results in real time [22]. Biosensors have been defined by the International Union of Pure and Applied Chemistry (IUPAC) as independent analytical devices that detect biological signals and convert them into measurable physical signals by using electrochemical-based, surface plasma-based, colorimetric-based, or surface enhancement Raman spectrum-based signals for detecting and demonstrating target molecules, and generating quantitative or semi-quantitative results [22,68]. The recognition element can be any bioreceptor, such as an antibody, enzyme, phage, nucleic acid probe, antigen, aptamer, whole cell, etc., that has been designed to specifically detect the target analyte, and used as a transducer for converting the biological or chemical signal into a measurable signal that can be read and analyzed [22,68,69]. Biosensors can be classified based on their signal transduction method into types such as electrochemical [70], optical [22], and piezoelectric devices [71]. Figure 4 demonstrates the basic design of a biosensor.

### 4.1. Electrochemical Biosensor

**Figure 5:**
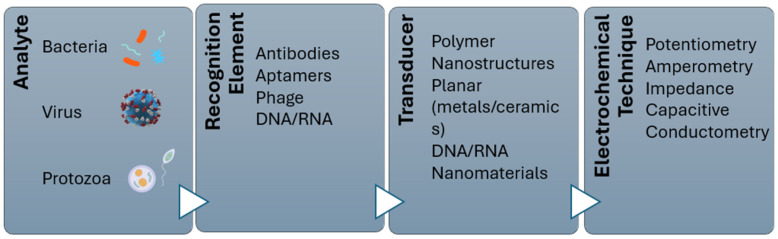
Key components of an electrochemical biosensor for bacterial detection. These include analyte (bacteria, protozoa, virus, etc.), biorecognition elements such as antibodies, oligonucleotides, phages, etc., transducer, and signal readout [26].

Among different types of transducers that have been studied for bacterial detection, including optical and mechanical, the electrochemical biosensor is the most common sensing technique applied for bacterial detection [70,73,74,75]. An electrochemical biosensor is a detection technology that is based on the principle of detecting electron transfer brought on by redox reactions or modifications to the electrodes’ surface properties [70]. Electrochemical biosensors have been extensively studied for different applications, including foodborne bacterial detection, driven by their advantages of low limit of detection, ease of use, miniaturization, robustness, ability to work with turbid samples, etc. [23,69]. They detect target analytes through electrodes by measuring electrical signals resulting from catalytic reactions or specific unions [69]. The electrical signals detection can be measured through techniques like potentiometry (different voltage measurement), amperometry (electrical current measurement), and electrical impedance spectroscopy (conductivity measurement) [66,76]. Their high detection sensitivity allows users to comply with food safety regulations and standards without running the danger of mass product recalls, and their fast detection response time makes it suitable for the demands of the food industry [77]. Figure 5 illustrates an electrochemical biosensor.

Various types of electrochemical biosensors have been developed and applied for *L. mono* and *Salmonella* detections, such as phage-based [70,77], antibody-based [73,74], nucleic acid-based [78], aptamer-based [78,79], cell-based [80], and nanomaterial-based electrochemical biosensors [81,82].

#### 4.1.1. Phage-Based Electrochemical Biosensors

Bacteriophages, also known as phages, are emerging as a promising tool for controlling foodborne pathogens. They have recently gained attention as excellent bio-recognition probes due to their unique ability to differentiate between dead and living cells, showing great potential for developing novel bacterial detection methods [51].

**Figure 6:**
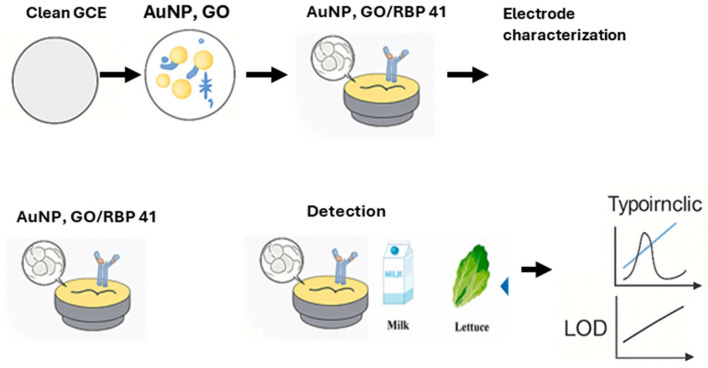
Schematic diagram outlining the preparation process for GCE modification with AuNPs, GO, and RBP 41, and the application of modified GCE (AuNPs, GO, and RBP 41) for the electrochemical detection of *S. Typhimurium* in food milk and lettuce food samples [71].

Ding and colleagues [71] have successfully developed a phage-encoded protein RBP 41-based electrochemical biosensor for specific and rapid detection of *Salmonella Typhimurium* (*S. Typhimurium*) in skim milk and lettuce food samples. They modified a glassy carbon electrode (GCE) with RBP 41, carboxylated graphene oxide (GO), and gold nanoparticles (GNPs) to capture *S. Typhimurium* using a differential pulse voltammetry (DPV) signal. For optimal electrode capture efficiency, key conditions such as *Salmonella* incubation time, GNPs deposition time, and RBP 41 concentrations were optimized. Figure 6 shows the schematic representation of the modification of the electrode and detection of *Salmonella* by a phage-based electrochemical biosensor.

**Figure 7:**
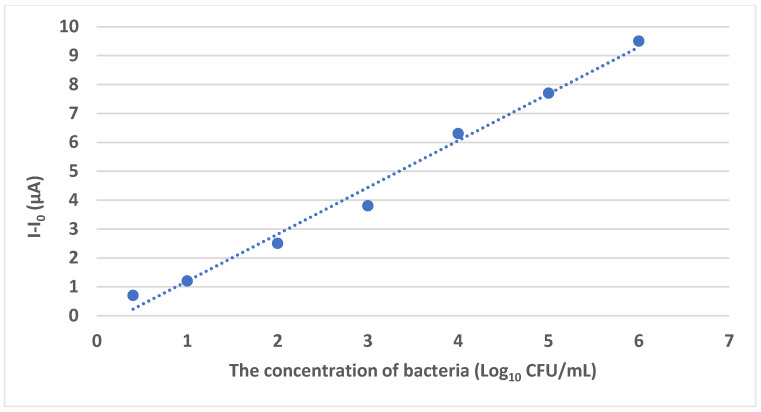
The linear fit curve for *Salmonella* detection by the RBP 41-based electrochemical biosensor (Y = 1.709x − 0.6685, R^2^ = 0.9976) [69].

The resulting biosensor could detect *S. Typhimurium* in low concentrations ranging from 3 to 10^6^ CFU/mL and a fast detection response time of 30 min, with a low detection limit of 0.298 Log_10_ CFU/mL. Moreover, the biosensor was also able to detect *S. Typhimurium* in the presence of other competing microorganisms such as *Escherichia coli* (*E. coli 0157:H7*), *Staphylococcus aureus*, *Vibrio parahemolyticus*, and *Pseudomonas aeruginosa*, including other species of *Salmonella* such a *Salmonella enteritidis*, *Salmonella paratyphi*, etc., demonstrating high specificity and sensitivity. Figure 7 shows the line fit curve for the detection of *Salmonella*, which demonstrates that the method can accurately detect *S. Typhimurium* with a correlation coefficient value of 0.997.

**Figure 8:**
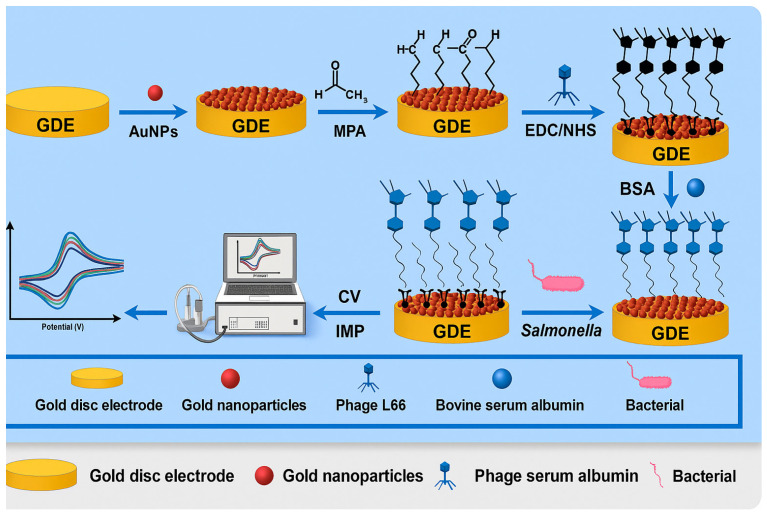
Illustration of the GDE-AuNPs-MPA-END/NHS-phage biosensor construction and *Salmonella* capture by the sensor using cyclic voltammetry [83].

In 2022, another study by Li and colleagues detected *Salmonella* using a phage L66 electrochemical biosensor. The *Salmonella* phage L66 used in the study as a bacterial capture was isolated from sewage by using *S. Typhimurium* as the host bacteria. The gold disc electrode (GDE) was modified through layer-by-layer assembly of AuNPs and 3-mercaptopropionic acid (MPA). Then, various concentrations of phage L66 solutions ranging from 2.5 × 10^3^ to 2.8 × 10^10^ PFU/mL were immobilized on the electrode’s surface (GDE-AuNPs-MPA) as shown by the schematic diagram of the method in Figure 8, and phage concentrations were measured.

The GDE-AuNPs-MPA-END/NHS-phage biosensor could quantitatively detect *Salmonella* in 3 different food matrices at different spike levels, as shown in Table 4. The sensor’s specificity was verified by co-culturing the sensor with different organisms, *Vibrio parahemolyticus*, *Escherichia coli*, *Staphylococcus aureus*, *Bacillus subtilis*, *L. mono*, and *various Salmonella serotypes* (*S. Indiana*, *S. enteritidis*, and *S. choleraesuis*), at the same concentration, 2.25 × 10^6^ CFU/mL. Significant changes in charge transfer resistance (R_CT_) were observed for *Salmonella* strain detection, while almost no increase in R_CT_ was observed for non-*Salmonella* strains, demonstrating the biosensor’s high specificity. In addition, these results were proven by the host spectrum test conducted in the study, which indicated the phage’s ability to specifically identify *Salmonella* host bacteria.

**Figure 9:**
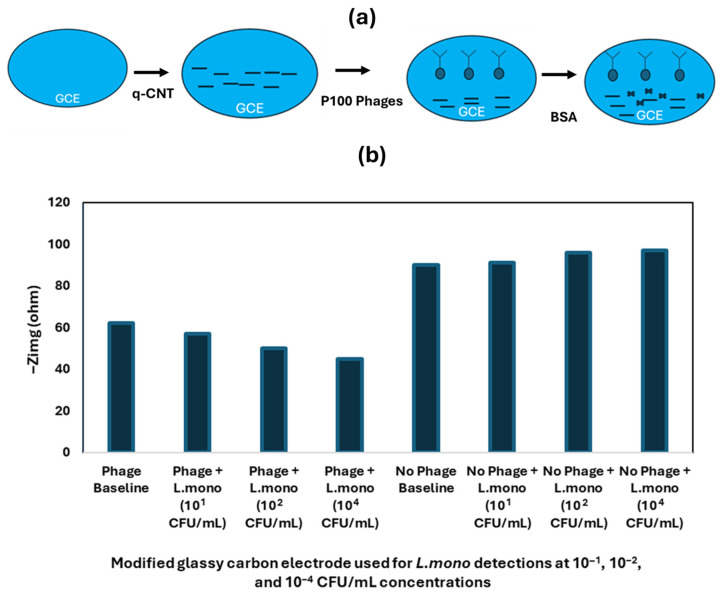
(**a**) Construction of the biosensor including electrode modification by q-CNT, PBSE crosslinker, P100 phage immobilization, and BSA. (**b**) Nyquist plot of the electrode after each modification [77].

**Figure 10:**
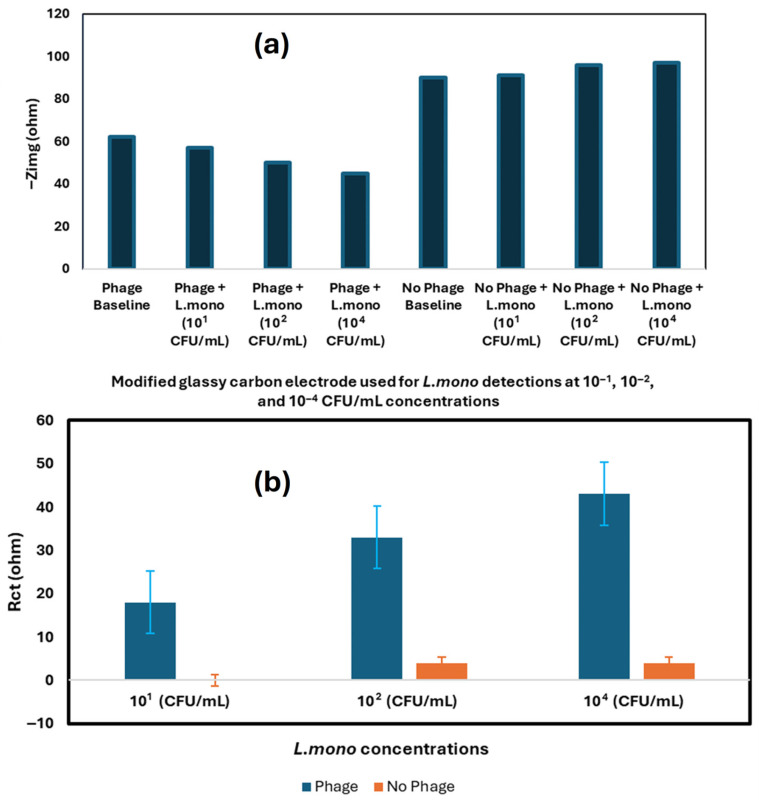
(**a**) Nyquist plots showing *L. mono* detections by the P100 phage-modified electrode and unmodified or bare electrodes at varying concentrations. (**b**) The graph showing the relationship between the differential charge transfer resistance and the logarithm of *L. mono* concentrations with and without the P100 phage [77].

Another phage-based electrochemical biosensor was developed by [77] for rapid *Listeria monocytogenes* (*L. mono*) detection using P100 phage as the bioreceptor. The biosensor performance was enhanced by using the carbon nanotubes functionalized with quaternized polyethyleneimine-modified CNT (q-CNT) to modify the electrode (GCE). The electrode was further modified through P100 phage immobilization to enable selection detection of the target bacteria. The P100 phage-modified electrode was used to detect *L. mono* in samples at various concentrations ranging from 1 CFU/mL to 10^8^ CFU/mL. Figure 9 illustrates biosensor preparation as well as electrode modification. The results of the biosensor for *L. mono* detection at different concentrations are shown in Figure 10.

*E. coli 0157:H7* and *S. Typhimurium* were used as non-target analytes to evaluate the biosensor’s specificity. The developed biosensor exhibited high sensitivity and specificity towards *L. mono*, with an 8.4 CFU/mL limit of detection as shown in Figure 10a and Figure 10b, respectively [77].

#### 4.1.2. Antibody-Based Electrochemical Biosensors

**Figure 11:**
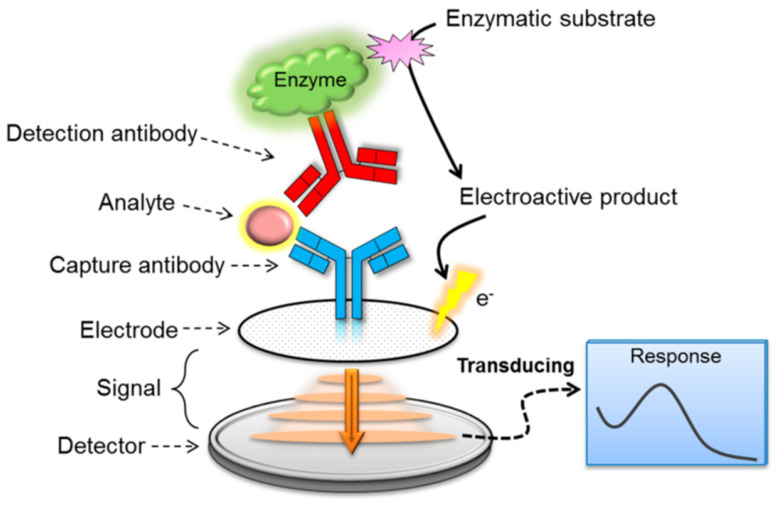
Schematic representation of a basic analytical principle of an electrochemical immunosensor. The unit is composed of the analyte that is passed over the sensor in solution and recognized by the biorecognition element, antibodies, through antibody–antigen interaction. The transducer generates a measurable signal based on changes in the biomolecule concentration. Data is analyzed on the computer [85].

An immunosensor, also known as an antibody-based biosensor, is the commonly used type of biosensor [22]. Immunosensors for bacterial detection require the specific interaction between antigens on the outer membrane of bacterial cells and specific antibodies on the carbon electrode. Antibodies bind to target analytes in complex biological mixtures with high selectivity and specificity and can detect molecules at relatively low concentrations [22,84], making immunosensors ideal for foodborne pathogens detection. The performance of the immunosensor depends on the type of antibody used and its immobilization on the electrode. The antibody determines its binding and has a great impact on the sensor’s sensitivity and specificity. For example, IgG antibodies are commonly used for heavy metals detection, while polyclonal antibodies are used for detecting other various antigen-binding sites [85]. Figure 11 shows a summarized representation of an electrochemical immunosensor.

**Figure 12:**
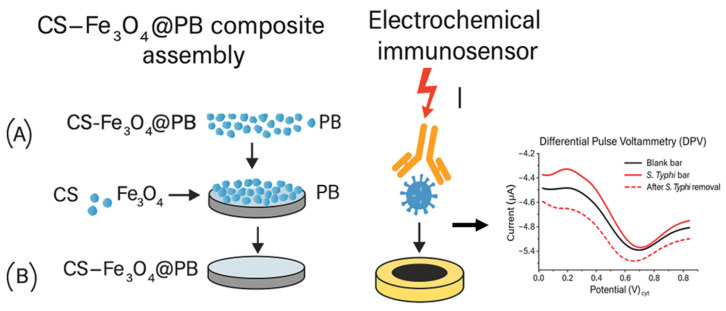
(**A**) Diagram illustrating CS-Fe_3_O_4_@PB composite assembly. (**B**) Electrochemical immunosensor design [73].

Yi and colleagues [73] developed an electrochemical immunosensor, using Fe_3_O_4_, to selectively and rapidly detect *Salmonella* in food. The immunosensor was constructed through a layer-by-layer assembly of Fe_3_O_4_@Prussian blue (PB), glutaraldehyde (GA), and *Salmonella* antibody on the gold electrode (GE), as shown in Figure 12. Then the modified electrode (BSA/Ab/GA/Fe_3_O_4_@ PB/GE) was immobilized in solutions/samples containing *Salmonella* at various concentrations. The Fe_3_O_4_@PB core–shell nanomaterial modified electrode was used as an internal signal, while [Fe(CN)_6_]^3−/4−^ ionic redox tags were used as external signals. The electrical signals were amplified using the synergistic effect of both internal and external signals. The detection performance of the immunosensor was assessed by detecting *Salmonella* at a range of concentrations under optimal conditions. A wide linear range of 7.375 × 10^1^ to 7.375 × 10^7^ CFU/mL, with a low detection limit of 9.912 CFU/mL, was achieved. According to the authors, the developed immunosensor has superior analytical performance and low limits of detection compared to other *Salmonella* detection methods reported in the literature.

**Figure 13:**
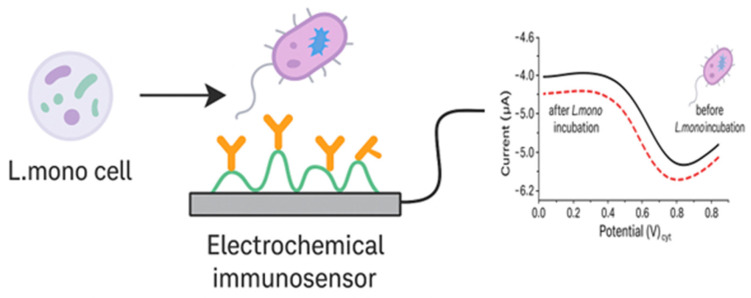
Representation of the *L. mono* cells detection by the electrochemical immunosensor and the detection system [74].

Another electrochemical immunosensor was developed by Cheng and colleagues [74] for rapid and sensitive detection of *L. mono* in milk. They cultured *L. mono* cells and used them to inoculate milk samples at various concentrations. The gold (Au) electrode was modified by immobilizing anti-*L. mono* antibodies, mouse monoclonal antibodies, on the electrode by self-assembled monolayers (SAM). The immunosensor was used to detect *L. mono* in milk at different concentrations, and *L. mono* was captured by the immobilized antibodies by specific recognition through antigen–antibody binding interaction. Then the second antibody, horseradish peroxidase (HRP)-labelled rabbit polyclonal antibody, was bound with the captured *L. mono* cells on the electrode, as shown in Figure 13.

**Figure 14:**
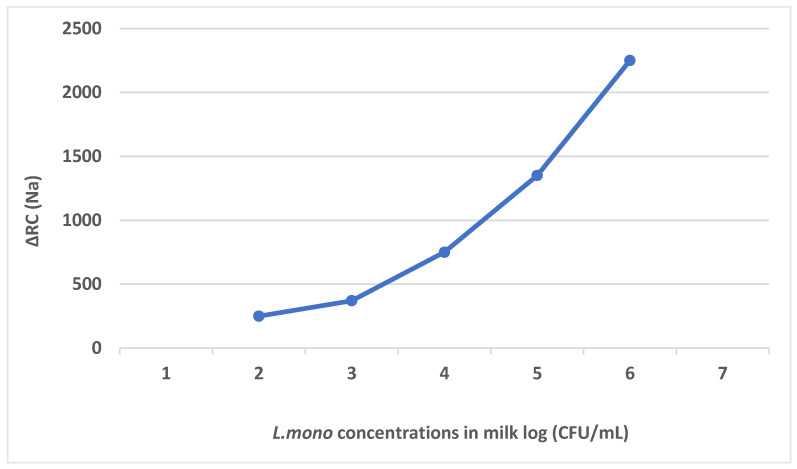
A direct relationship between the concentrations (1.0 × 10^2^ to 1.0 × 10^6^) of *L. mono* in milk samples with the response current (y = 568.6x − 1417.2, R^2^ = 0.9693) [74].

A direct linear correlation was observed between the response current and the logarithm of *L. mono concentrations*, from 1.0 × 10^2^ to 1.0 × 10^6^ CFU/mL, as shown in Figure 14, proving the specificity of the biosensor as well as the ability to accurately identify *L. mono*. These results were also compared to the other results obtained from the plate count method, and consistency was observed.

#### 4.1.3. Nucleic Acid-Based Electrochemical Biosensors

Nucleic acid-based biosensors, known as genosensors [86,87], have become increasingly popular for detection due to their high specificity, sensitivity, rapidity, simplicity, and affordability, making them a promising technology for detecting bacteria [66,78,88]. They use short single-stranded DNA and RNA probes as receptors to bind to specific targeted DNA or RNA sequences through hybridization, enabling detection [66].

Clustered regularly interspaced short palindromic repeats (CRISPR)-associated (Cas) system has emerged as a powerful tool for detecting nucleic acids [78]. Zheng and team (2023) successfully invented a ratiometric electrochemical biosensor integrated with the saltatory rolling circle amplification (SRCA) and CRISPR/Cas12a system for ultrasensitive and specific *Salmonella* detection in 50 food matrices, pork, beef, mutton, donkey, dairy, and RTE egg products, collected from local markets. They designed a pair of primers to initiate the SRCA reaction based on the invasion gene of *Salmonella*, *invA* gene. They then devised the crRNA to specifically identify the target DNA based on the target’s protospacer adjacent motif (PAM) sequence. When the guide RNA recognizes the target DNA, it activates the Cas12a enzyme trans-cleavage activity, allowing successful ssDNA reporter cleavage.

They used a three-electrode system which consisted of GCE as the working electrode, a counter electrode of platinum wire, and a reference electrode of Ag/AgCI. The GCE was modified through immobilization into AuNPs and Fc-hp solutions, creating Fc-hp/AuNPs/GCE for performance enhancement. FAM-ssDNA-BHQ1 reporter was introduced into the system for fluorescence detection of *trans*-cutting activity of Cas12a/CrRNA. A rapid increase in the fluorescence intensity was observed in the presence of the target DNA-Cas12a-crRNA complex, while a low fluorescence signal was observed in the absence of the target DNA, Cas12a, or crRNA, demonstrating accurate recognition of the SRCA products by crRNA and the Cas12a *trans*-cutting activity stimulation to cleave the ssDNA reporter [78].

The resulting biosensor successfully detected *Salmonella*, demonstrating a linear detection range spanning from 5.8 fg·µL^−1^ to 5.8 ng·µL^−1^, and 2.08 fg·µL^−1^ LOD for *S.typhi*. The biosensor also demonstrated high specificity when detection was performed on non-*Salmonella* strains, *L. mono*, *E. coli*, *C. sakazakii*, *S. aureus*, and *S. flexneri.*

**Figure 15:**
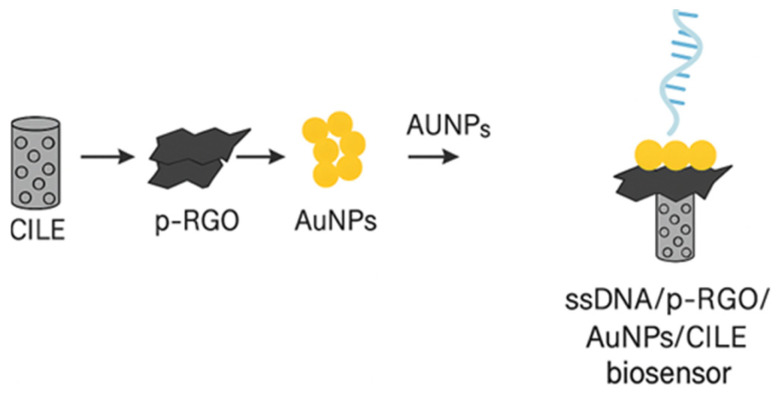
Construction of ssDNA/p-RGO/AuNPs/CILE biosensor [88].

Another electrochemical DNA sensor for *L. mono* detection was built by [88]. The biosensor was targeting the *L. mono hly* gene sequence. They modified the carbon ionic liquid electrode (CILE) with gold nanoparticles and partially reduced graphene oxide (p-RGO). The modified electrode was then covalently bound to the probe ssDNA, forming ssDNA/p-RGO/AuNPs/CILE biosensor as shown in Figure 15.

Under the optimal conditions, the designed biosensor was used to detect *L. mono hly* gene sequence in the concentration ranging from 1.0 × 10^−13^ to 1.0 × 10^−6^ mol/L. The biosensor achieved 3.17 × 10^−14^ mol/L (3S_0_/S) LOD and showed good sensitivity, stability, and ability to effectively detect *L. mono hly* gene sequence PCR products in meat samples, demonstrating the DNA sensor’s real application. Furthermore, the sensor’s selectivity was performed through hybridization of the probe ssDNA modified p-RGO/AuNPs/CILE with different ssDNA sequences, one-base mismatched, three-base mismatched, non-complementary, as well as the target ssDNA sequence. The sensor showed high selectivity to various ssDNA sequences.

#### 4.1.4. Aptamer-Based Electrochemical Biosensors

**Figure 16:**
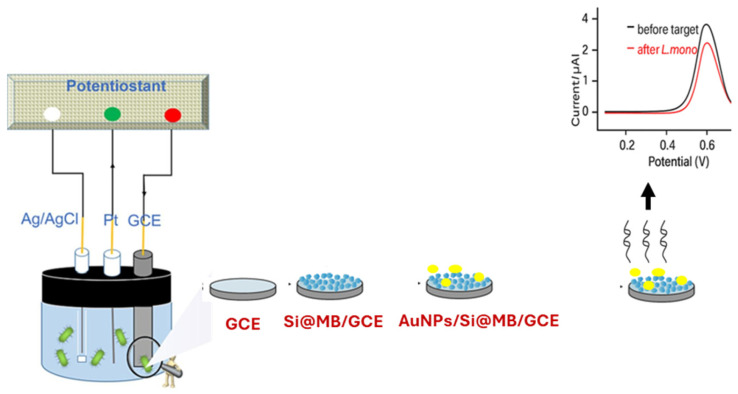
*L. mono* detection using the Apt-AuNPs/Si@MB/GCE electrochemical biosensor [90].

Aptamers are short nucleic acids or peptide chains that bind specifically to a target [89]. Zhang et al. [90] (2023) developed an aptamer-based electrochemical biosensor for *L. mono* in lettuce and fresh-cut fruits in just 1.5 h. Three-electrode system was used, GCE as the working electrode, platinum wire as the counter electrode, and Ag/AgCI as the reference electrode. The GCE was sequentially modified with methylene blue (Si@MB), AuNPs, and an aptamer (Apt), as shown in Figure 16, for better bacteria detection.

The samples, lettuce and fresh-cut fruit, were contaminated with varying *L. mono* concentrations, 10^4^ to 10^7^ CFU/mL. The Apt-AuNPs/Si@MB/GCE biosensor was used to detect *L. mono* in various concentrations (1.6 × 10^8^, 1.6 × 10^7^, 1.6 × 10^6^, 1.6 × 10^5^, 1.6 × 10^4^, 1.6 × 10^3^, 1.6 × 10^2^, 16 CFU/mL), and this was carried out using DPV.

The biosensor was able to detect *L. mono* in samples containing *L. mono* via specific recognition of the aptamer. The biosensor achieved good linearity, ranging from 10^−2^ to 10^−7^ CFU/mL, with an LOD of 2.6 CFU/mL. In addition, the biosensor also successfully detected *L. mono* in real samples, with the recoveries ranging from 80.0% to 110% in fresh-cut fruit and from 84.7% to 116.0% in lettuce. In addition, the relative standard deviation (RSD) range of 3.8–4.5% and 3.2–4.7% also verified the biosensor accuracy and sensitivity in real-life applications. The developed biosensor was also able to selectively detect *L. mono* in samples containing *S. aureus* and *Salmonella* at high concentrations, demonstrating high selectivity. Lastly, the sensor’s good reproducibility was also observed, with the mean RSD of 3.7%.

#### 4.1.5. Cell-Based Electrochemical Biosensors

Cells and their components are being used to develop advanced biosensors and biochips. This type of biosensor utilizes cell organelles as bioreceptors [91] and has a more outstanding performance. Cell-based biosensors use living cells to detect changes in their environment, responding to external stimuli or environmental changes. When external stimuli are detected by cells, the signal that is generated by molecular recognition and cell signal transduction goes through physical or chemical transducers. Then the signal is converted into an electrical signal that can be measured and analyzed [92].

**Figure 17:**
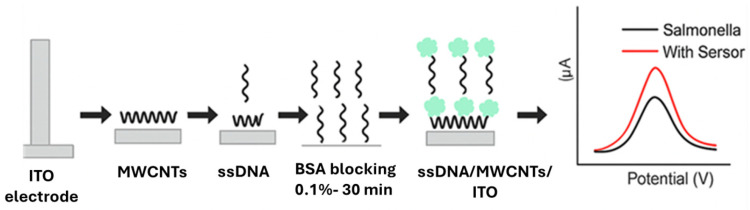
The development of ssDNA/MWCNTs/ITO electrochemical biosensor for *Salmonella* detection [80].

Hasan and colleagues [80] (2018) developed a multi-walled nanotube (MWCNTs)-based aptasensor for sensitive electrochemical detection of whole-cell *Salmonella*. To optimize their sensing tool, they modified the indium tin oxide (ITO) electrode through immobilization into activated MWCNTs/ITO and amino-modified *Salmonella* DNA aptamer solutions, forming ssDNA/MWCNTs/ITO, as shown in Figure 17.

**Figure 18:**
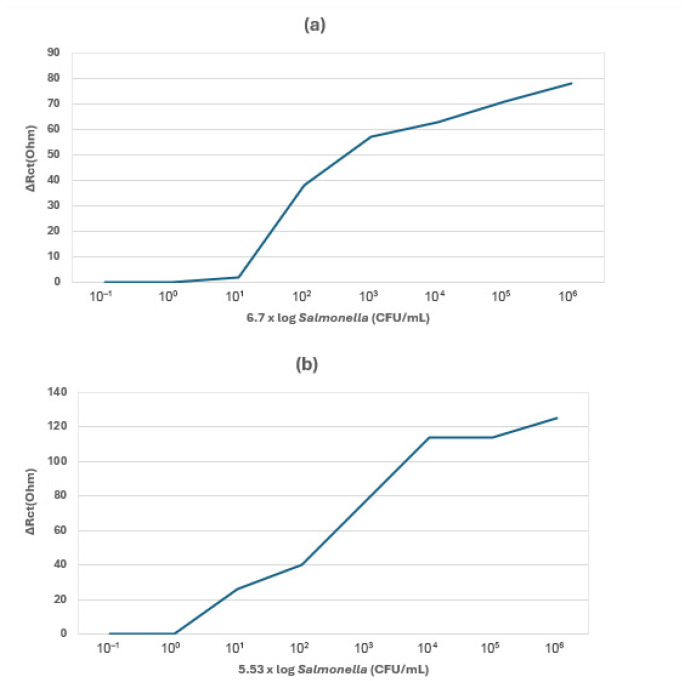
Line graphs analysis for (**a**) *S. Typhimurium* detection in 5.5 × 10^1^ to 5.5 × 10^6^ CFU/mL concentrations (Y = 11.637021X + 14.52964, R^2^ = 0.91691), and (**b**) *S. enteritidis* in 6.7 × 10^1^ to 6.7 × 10^5^ CFU/mL concentrations (Y = 17.05321X + 27.99468, R^2^ = 0.95787) [80].

The modified electrode was incubated in different concentrations of *S. enteritidis* (6.7 × 10^1^ to 6.7 × 10^5^ CFU/mL) and *S. Typhimurium* (5.5 × 10^1^ to 5.5 × 10^6^ CFU/mL), respectively. LODs of 5.5 × 10^1^ CFU/mL for *S. enteritidis* and 6.7 × 10^1^ CFU/mL for *S. Typhimurium*, determined from the sensitivity analysis, were obtained, as shown in Figure 18. The sensitivity of the aptasensor was validated using PCR through *invA* gene detection. PCR limit of sensitivity of 10^2^ CFU/mL was found to be higher than that of the developed aptasensor (10^1^ CFU/mL), proving the aptasensor’s high sensitivity.

Furthermore, the aptasensor’s real-life application was evaluated by conducting detections in different raw chicken samples purchased from the market in parallel with the conventional method. The aptasensor achieved 10^1^ CFU/mL LOD in all three samples, and its results were validated by the culture-based method and PCR targeting the *invA* gene. This demonstrated the aptasensor’s suitability to detect *Salmonella* in real food samples. In addition, the biosensor’s specificity was evaluated by adding five *Salmonella* serovars, *S.typhi*, *S.typhimurium*, *S.paratyphi*, *S.enteritidis*, *S. paratyphi* A *and S.paratyphi* B, and non-*Salmonella* strains, *Vibrio parahaemolyticus*, *S.aureus*, and *E.coli*, and the aptasensor showed no specificity to non-*Salmonella* bacteria and high specificity to *Salmonella*. Lastly, it is also worth noting that the application of this type of biosensor and ferricyanide/ferrocyanide, as an aptasensor for *Salmonella* detection, has never been reported before.

#### 4.1.6. Nanomaterials in Enhancing Electrochemical Biosensor Performance

**Figure 19:**
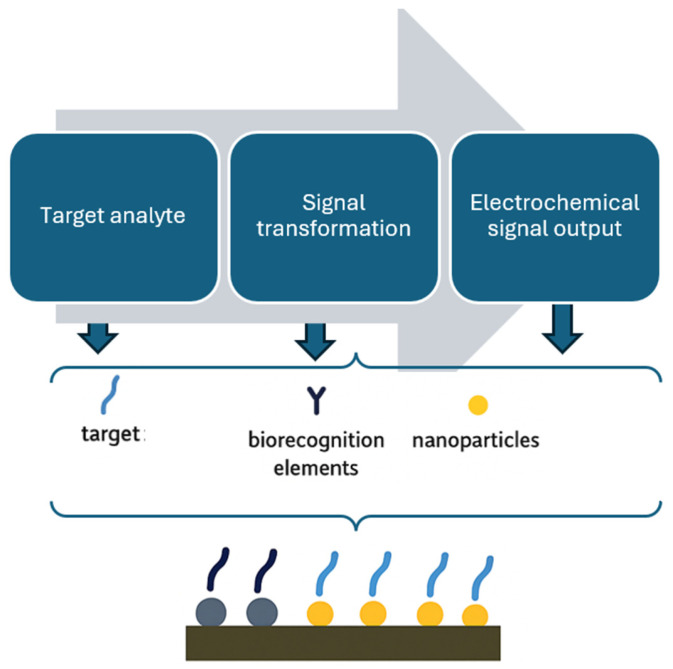
Schematic representation of nanoparticle-based electrochemical biosensors [93].

Nanomaterials are commonly used in electrochemical biosensors to enhance and improve the biosensor’s performance [80]. Magnetic nanoparticles, quantum dots, and carbon nanostructures promote fast electron transfer, high sensitivity, and lower LOD. Graphene and metal nanoparticles are commonly used to increase the electrochemical biosensor’s surface area and conductivity. Additionally, adding metal nanoparticles to the working electrode enhances the electrochemical biosensor’s sensitivity and signal response time [87,93]. Nanomaterials-based electrochemical biosensor is shown in Figure 19.

Zolti et al. (2024) [82] investigated the efficiency of a biosensor for *L. mono* detection in chicken broth samples. They used a three-electrode screen-printed electrode (SPE) electrochemical biosensor system consisting of working, counter, and reference electrodes. The SPEs were modified with the quaternized carbon nanotubes (q-CNT) to improve the biosensor’s performance, followed by the addition of the Listex P100 bacteriophage (P100 Phage) as a bioreceptor. Finally, the modified SPEs were tested in phosphate-buffered saline (PBS) buffer containing *L. mono* at a concentration range of 10^2^ to 10^6^ CFU/mL and diluted chicken broths. The negative control (containing no bacteria) served as the baseline R_CT_ for each set of measurements. Table 5 demonstrates the recovery rates in buffer and chicken broth.

The results presented in Table 5 prove the biosensor’s predictability and accuracy. The sensor’s performance improved with higher concentrations in broth samples, and in some concentrations, showing better results than when in PBS buffer. This shows the biosensor’s ability to detect *L. mono* and produce a reliable measurement of its concentrations in chicken broth samples, with 88–96% T accuracy. The biosensor achieved a 10 CFU/mL LOD in chicken broth and a 55 CFU/mL LOD in PBS buffer. For specificity, ser. *Typhimurium—291RH* and *E. coli* 0157 were used as non-targets, and the biosensor showed high specificity for *L. mono*.

**Figure 20:**
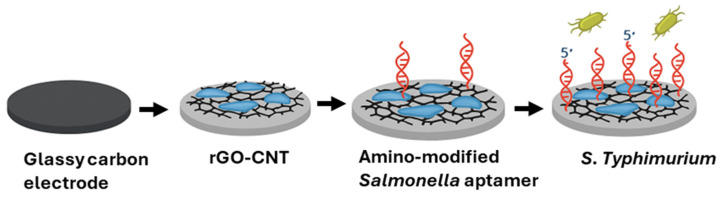
Stepwise glassy carbon electrode modification by rGO-CNT and amino-modified *Salmonella* aptamer (ssDNA/rGO-CNT/GCE aptasensor) for electrochemical detection of *S. Typhimurium* [81].

Another nanomaterial-based biosensor for rapid and sensitive detection of *S. Typhimurium* in five raw chicken meat samples was designed by Appaturi and team (2020). The detection was performed using a reduced graphene oxide-carbon (rGO-CNT) based electrochemical apatasensor. The glassy carbon electrode was modified through rGO-CNT suspension, followed by amino-modified *Salmonella* aptamer, forming ssDNA/rGO-CNT/GCE aptasensor. Figure 20 shows the overall modification of the glassy carbon electrode. The culture-based method was used to validate the results of the sensor.

Five chicken samples tested positive for *Salmonella* in various *Salmonella* concentrations ranging from 10^1^–10^3^ CFU/mL^−1^, while responses below LOD were obtained for C4 and CS5. The results obtained using the aptasensor were comparable to those obtained using the culture-based method, demonstrating the applicability of the aptasensor in food analysis. Furthermore, the developed aptasensor successfully detected *Salmonella* in the presence of other non-target bacterial cells, showing high specificity. LOD of 10^1^ CFU/mL was achieved. A summary of all the biosensing approaches discussed above has been tabulated in Table 6.

## 5. Comparative Performance of Detection Methods

Different studies investigated the detection of *Salmonella* and *L. mono* in food matrices using various methods, including culture-based, culture-independent, spectroscopy-based, and electrochemical biosensors [11,63,70]. Table 7 shows the comparison of the methods used for *Salmonella* and *L. mono* detection in food matrices at different concentrations.

## 6. Challenges and Future Perspectives of Electrochemical Biosensors

**Figure 21:**
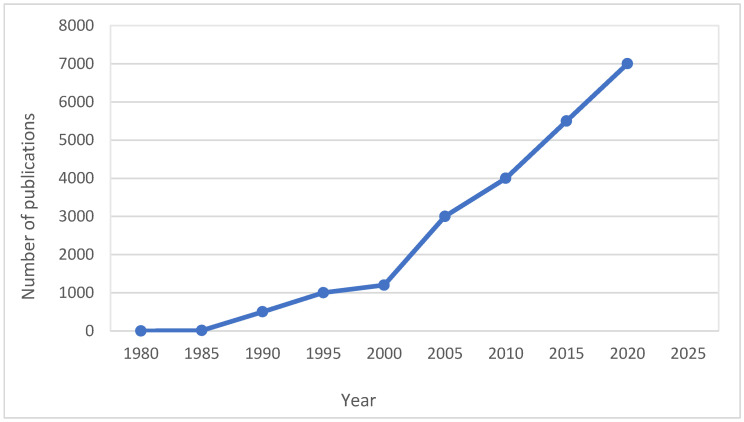
The number of papers published on biosensors from 1980 to 2022 [71].

There is an increasing number of papers on the application of biosensors in the food industry. A 6.65% annual growth of electrochemical biosensors has been reported, with an expectation of the market reaching USD 26.8% billion by the end of 2030 [72]. Growing interest in the application of biosensors, based on the number of publications, is shown in Figure 21.

Despite the increase in research focused on the development of biosensors that are rapid, specific, and sensitive for pathogen detection, biosensors present some limitations. Electrochemical biosensors have been successfully developed and used by many researchers all over the world for many applications [78,80,82]. While its application in foodborne bacterial detection is promising, it is faced with several challenges, including:

- Commercialization: Electrochemical biosensors are facing commercialization challenges due to short device lifespan and lack of unified standards and regulatory guidelines [71,91,94].
- Environmental stability: Fluctuations in humidity, temperature, and ambient light conditions may affect the sensor’s performance [95].
- Nanomaterials: It is still difficult to achieve perfect carbon nanomaterials and biological elements immobilization to enhance the electrochemical biosensor’s detection performance [96].
- On-site detection: Development of miniaturization electrochemical biosensors for on-site pathogen detection is still a challenge [97].

### 6.1. The Matrix Effect

The matrix effect is recognized as one of the critical factors that influence trueness in analytical measurements, and it is more important in the analysis of critical compounds [98]. Matrix effects can significantly affect the accuracy, sensitivity, and reliability of biosensing technologies, presenting a formidable challenge to the reliability of the results. In complex matrices such as foodstuffs, wastewater, soil, biological samples, etc., other components such as molecules in the matrix can interact with the analyte, interfering with the measurement. Additionally, matrix can also interact with the sensor surface and depending on the specificity of the matrix or molecules in the matrix, and whether the sensor is mass or refractive index sensitive, this can affect the sensor’s performance, leading to a significant change in the response of the sensor (drift) [99].

It is important to address matrix effects to achieve accurate and precise measurements in complex matrices. The multifaceted nature of matrix effects can be influenced by a variety of factors, such as target analyte, sample preparation protocol, and composition [100]. For electrochemical biosensing, matrix effects can lead to the following: (1) electrode fouling by proteins/lipids, ionic strength (2) changes altering electron transfer, (3) adsorption of redox-active interferents, and non-specific (4) adsorption blocking biorecognition.

(1) electrode fouling by proteins/lipid

Biofouling of a biosensor is typically linked to the adsorption of proteins, cells, bacteria, etc., to the electrode surface; for biosensors that are then implanted, such fouling can further stimulate downstream foreign body reactions or immune reactions [2]. In electrochemical biosensing, complex matrix components like proteins, cells, lipids, and analytes, as well as side products of the electrochemical reaction, can attach to the surface, preventing the target analyte from attaching to the biorecognition element and/or impeding electron transfer from/to the electrode surface [3]. Such fouling in turn passivates the electrode and leads to a higher background signal, lower sensitivity, and thus lower signal-to-noise ratios that compromise the detection limit and shorten the shelf-life of electrodes exposed to physiological fluids [4].

A variety of strategies have been proposed to counter biofouling in electrochemical sensor systems, with many reported approaches involving surface modifications that render the surface more hydrophilic, electrically neutral, flexible, and/or hydrogen bond accepting to suppress the non-specific interaction of biofoulants with the surface [5]. Most commonly, gold electrochemical biosensors use hydroxyl group-terminated small thiol molecules such as mercaptohexanol (MCH), mercaptoethanol, or mercaptoundecanoic acid as a backfiller in tandem with thiolated capture probes as biorecognition elements for reducing the interaction of biofoulants with the electrode surface [6].

(2) changes altering electron transfer

Sensitivity is a key consideration for many food detection technologies, because many food pathogens are present at nanomolar to picomolar concentrations, and the biosensor must achieve sufficient sensitivity in a complex matrix of interferent molecules [7]. Unfortunately, due to noise limitations in existing electronic measurement systems, the signal-to-noise ratio of conventional electrochemical biosensors degrades precipitously when they are miniaturized to the micron scale, reducing their sensitivity and making meaningful measurements of analyte concentrations challenging or even impossible in many cases [8].

There have been several advances in the fabrication of nanostructured electrodes over the last decade, which have achieved improved sensing properties relative to standard planar electrodes, such as increased signal levels and faster diffusion of redox species [9,10,11]. In a seminal study, Kelley and co-workers demonstrated that nanostructured electrodes with high surface curvature, which they termed “nanoflowers,” can greatly enhance DNA detection compared to planar electrodes, with limits of detection (LOD) in the femtomolar range. Seker and coworkers have shown that similar improvements in sensitivity can also be achieved with nanoporous electrodes, with the additional benefit that the sensitivity and dissociation constant (*K*_D_) of the resulting sensors can be tuned by changing the size of the nanopores [12].

**Figure 22:**
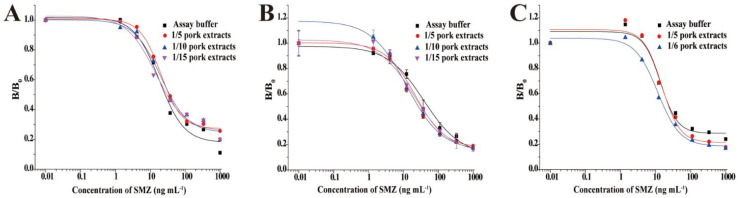
(**A**) Pork matrix effect with different dilution ratios, (**B**) pork matrix effect (fat removed) with different dilution ratios using PBS containing 5 mM MgCl_2_ as extraction buffers, and (**C**) pork matrix effect with different dilution ratios of PBS containing 5 mM MgCl_2_ and 0.1% of BSA [100].

Furthermore, matrix effects can be tackled by improving or implementing sample preparation and extraction procedures that minimize or eliminate the coextraction of interfering matrix effects. Dilutions of sample extracts and/or modifications of reagent concentrations, such as extraction buffers, have become one of the most used techniques in biosensing for reducing interfering compounds in complex samples [100]. Liang and colleagues, 2020 [101], developed a dihydropteroate synthase (DHPS)-based biosensor for the detection of multi-sulfonamides (SAs) in different food matrices. For quantification of matrix interferences, a standard curve obtained in assay buffer was compared with a calibration curve obtained in the sample matrix. If the two curves could be superimposed, the effect of the matrix was not significant, and vice versa, and the sample could be analyzed according to the calibration curve prepared in the assay buffer. Figure 22 shows the matrix effect of pork with different dilution ratios using PBS as the extraction buffer.

As shown in Figure 22, the standard curves produced in assay buffer and pork extracts (fat removed) after 5-, 10-, and 15-fold dilutions with PBS (containing 5 mM MgCl_2_) could not be superimposed (Figure 22B), indicating that the buffer used did not sufficiently remove matrix effects. After that, a 0.1% BSA was added to PBS (containing 5 mM MgCl_2_), and an obvious improvement was observed. They then used the PBS containing 5 mM MgCl_2_ to dilute the supernatant liquid layer 1:5. This procedure successfully reduced the matrix effects, allowing for the detection of the target analyte with relatively low LOD, 20.55 μg kg^−1^, as shown in Figure 22C.

In addition, the quick, easy, cheap, effective, rugged, and safe (QuEChERS) method has also been reported as one of the techniques that can be used to combat matrix effects while ensuring adequate analyte recovery, particularly in the analysis of complex matrices such as food, biological, and environmental samples. This method functions as an efficient sample preparation and clean-up technique by removing unwanted matrix interferences, providing clean and interference-free extracts [100].

**Figure 23:**
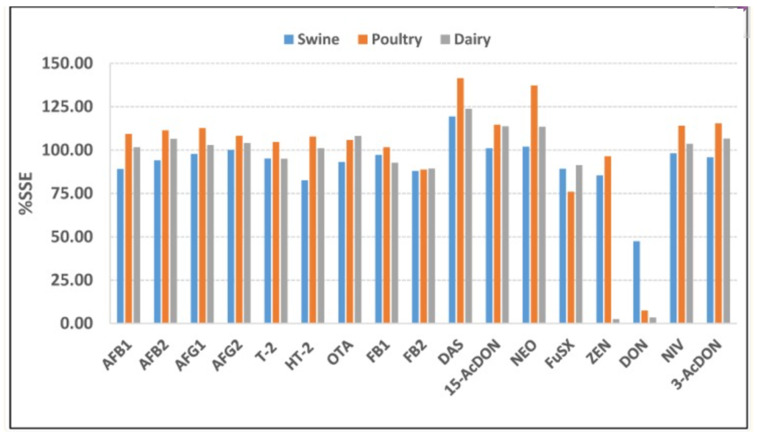
Signal suppression/enhancement (%SSE) for mycotoxin comparison between matrix-matched and solvent calibration using isotopically internal standard (ISTD) for three sample matrices. Additionally, preconcentration techniques such as immunomagnetic separation or aptamer-functionalized beads followed by a wash step have proved especially effective to both remove co-extractives and increase analytical sensitivity [102].

Another approach to tackle the matrix effect is by using signal suppression/enhancement (SSE), which represents the percentage change in analytical signal caused by co-extractives in the sample matrix, relative to the signal obtained in a clean solvent, as shown in Equation (1).

(1) $$SSE\%=\left(\frac{{Slope}_{Matrics}}{{Slope}_{Solvent}}\right)\times 100$$

where SSE values near 100% indicate negligible matrix effects, values < 100% indicate suppression, and values > 100% indicate enhancement. The study by Nualkaw [102] et al. used %SSE to evaluate the matrix effects in the three types of feed matrices. If the suppression or enhancement was marginal, the %SSE would be very close to 100%; if there was strong suppression/enhancement, the %SSE would deviate from 100%. In the swine feed samples, the %SSE was in the range 82.5–119.3%, except for DON, which exhibited strong signal suppression with the %SSE less than 50% (47.4%). In the poultry feed samples, the %SSE was in the range 76–115.4%, except for DON, which exhibited strong signal suppression (%SSE 7.49%), with strong signal enhancement for NEO and DAS (%SSE 137% and 142%, respectively). In the dairy feed samples, the %SSE was in the range 89.3–113.7%, except for DON and ZEA, which produced the same results as for the poultry feed samples, namely strong signal suppression with %SSE 2.5% and 3.5%, respectively. Regarding signal enhancement, the %SSE was greater than 120% (123.8%) for DAS. The %SSE values of the three types of feed matrices are summarized in Figure 23.

### 6.2. Bacterial Adaptive Response

Bacterial adaptive responses also present another major challenge. Over time, bacteria have evolved sophisticated defence mechanisms that today protect them against various threats such as bacteriophages, competing bacteria, and predators [100,101]. The capacity to detect and respond to surrounding changes is fundamental for their survival or growth in changing and extreme environments, such as variations in chemical concentrations, pH levels, and temperature [102,103]. Some bacteria have shown the ability to develop stable resistance against adverse conditions within a defined genomic context through the process called stress tolerance responses. This process includes both structural and physiological modifications within the bacterial cells, such as DNA, proteins, lipids, etc. [103]. These adaptive responses present difficulties in bacterial detection and may significantly influence the accuracy of the biosensor, as target molecules and bacterial metabolism may be altered during adaptation [100,103].

The accuracy, reproducibility, and selectivity of the biosensor depend fundamentally on the precise molecular recognition of the target, as biosensors rely on highly specific interactions between the biological recognition element and the target molecule [22,69,104]. Any significant change in the recognition of a target molecule, such as chemical composition, charge, shape, etc., due to bacterial adaptive response, may lead to the sensor’s low selectivity, non-specific binding, and reduced binding affinity.

### 6.3. Challenges of Sample Preparation and the Need for Lab-on-a-Chip Biosensor

Lab-on-a-chip (LOC) biosensor has been investigated and developed as a potential replacement for traditional and centralized laboratory testing [105,106]. It is multidisciplinary as it involves integration of different technologies and subject areas such as engineering, biophysics, molecular biology, computing science, chemistry, biochemistry, as well as the environmental sciences to enable sample preparation, analyte separation, and target detection in miniaturized formats, providing testing away from specialized centralized laboratory [107]. However, this field is presented with many challenges, with sample preparation/pre-treatment being a major one. The complex nature of food sample matrices makes the process of sample preparation/pre-treatment difficult, making the application of biosensors outside laboratory settings difficult and seemingly almost impossible [108]. LOC biosensor challenges include:

- Sample preparation—Sample preparation is difficult, especially in fruits and vegetables, as they need to be ground and filtered or centrifuged for the removal of large debris such as tissue fragments and plant cells. This process requires multiple centrifuging, pellet resuspension, cell lysis, etc., for improved performance. It is challenging to minimize and integrate these complex procedures into one or two simple steps using only small and basic equipment to allow the operation of the sensor by non-experts with minimal processing time [105].
- Limited microfluidic biosensor sensitivity—This is due to the very small sample volume of less than 100 μL that is often used. The presence of many pathogens is not allowed in many food products, including agrifoods, that is, at least 1 CFU/mL sensitivity is required [109].
- Reagents’ addition—The process of adding reagents to a chip requires human operation, which may increase interference and complexity.
- Less integration of food sample loading and biosensing signal readout with magnetic separation and biosensor—Although Xue et al. [109], have reported in their review great achievements in the integration of magnetic separator and biosensor onto a single chip by many authors, food sample loading and biosensing signal readout still need to be further integrated with a magnetic separator and a biosensor to achieve complete pathogen testing, without potential cross contamination [109].

The team further reported the design of a “LOC” biosensor that has achieved sample-in-answer-out detection of *Salmonella* with 350 CFU/mL LOD. According to the data published by [110], this sensitivity is too low for food pathogen detection. Xue and team [109] have also reported the *L. mono* detection lab-on-a-chip technique that was performed where an estimated 10 CFU/mL LOD was achieved. According to the data published by Zolti and colleagues, 2023, <10 CFU/mL *L. mono* is fit for human consumption. So, a LOC biosensor with a lower LOD is ideal.

Research in the microfluidic electrochemical biosensor has moved to the development of fully integrated LOC analytical systems that can combine all steps necessary for bacterial detection, from sample preparation to detection. Another future goal is to develop LOC with multiplexing capabilities to process large volume samples and to screen multiple pathogens simultaneously. Lastly, in-house testing by the food industry can be achieved by integrating LOCs with a smartphone-based user interface for easier adoption [110].

The technology for detecting individual pathogens is laborious, expensive, and time-consuming. Despite the increase in the emergence of electrochemical biosensors for foodborne bacterial detection, the development of novel methods and improvements to achieve the multianalyte detection of bacteria is required. The discovery of more pathogens increases the demand for multi-pathogen detection techniques. It has already been reported that the development of biosensor devices capable of detecting different pathogens, viruses, and bacteria without labeling is underway. This research is primarily focused on the design of different electrodes specific to bacteria [111,112].

On that note, among many other authors, Kaci and colleagues, 2023, [113] have broadened the field by successfully developing an easy, rapid, sensitive, and multiplex electrochemical DNA biosensor for specific and simultaneous detection of *L. mono* and *Salmonella* DNA sequences, with the application of nanomaterials. Wu et al., 2025, [112] also conducted a systematic review on the progress of research in recent years on optical biosensors for multiplex pathogenic detection, including *L. mono* and *Salmonella*. In their review, they have reported the incredible work performed by many different authors on multi-detection of various bacteria and viruses, which presents a promising development in the field of multiple biosensing.

In response to the increasing global emphasis on economic, environmental, and social responsibility, new remediation technologies are being developed to promote a more sustainable future. The term “green” refers to the advancements of electrochemical biosensing technologies that use biodegradable and sustainable materials, reducing the use of harmful/hazardous reagents and solvent systems. Paper has been considered the most promising substrate for the development of electrodes due to its low cost, and because it can be easily disposed of by incineration, it is safe for handling, etc. Biowaste-based sensors are also an emerging, environmentally friendly sensing platform as they utilize inexpensive and readily available biowaste materials to develop sensitive electrode surfaces, assisting with waste treatment and recycling. On the other hand, deep eutectic solvents (DESs)-based sensors have been used to replace conventional solvents due to their environmental friendliness, biodegradability, non-toxicity, and low cost [114,115].

In addition to the above-mentioned challenges, it was also discovered during this study that there is not enough literature on electrochemical immunosensors and RNA-based electrochemical biosensors for *L. mono* and *Salmonella* detection in food matrices.

It is believed that with continuous research, the above-mentioned challenges will be effectively solved. Future developments and recommendations include the development of methods that could simplify biosensor development, improve the practical application of nanomaterial-based electrochemical biosensors, and focus on developing biosensors that are able to operate on all food matrices and at different concentrations. In addition, carbon nanotubes have excellent electrochemical properties and also open the possibility of combination with other technologies in the future, for better and improved foodborne pathogens detection. Another future advancement includes the integration of biosensors with digital platforms and the establishment of uniform protocols for biosensor fabrication and performance assessment. The integration of CRISPR technology into biosensors also shows promise for the highly sensitive and rapid detection of pathogenic bacteria in food matrices, highlighting its potential in food safety monitoring. Lastly, detection of live bacteria is also vital; more research on metabolic-activity-based and cell-membrane-based biosensors is required to overcome this limitation [116].

## 7. Conclusions

Foodborne bacteria remain a major public health problem worldwide. *Salmonella* and *L. mono* are among the leading agents of foodborne diseases and outbreaks. These microbes are found in many various products, from raw to cooked. Therefore, their quick and accurate analysis in food matrices is of great importance to ensure public food safety. Culture-based, culture-independent, and spectroscopy-based methods are faced with limitations. Therefore, more biosensor development, in particular, electrochemical biosensors, is needed for better analysis and to overcome these limitations.

Electrochemical biosensors are good alternatives to molecular detection methods due to their ease of use, quick response, high specificity, and sensitivity. Several authors have proved that through their successful development of various biosensors for *Salmonella* and *L. mono* detection in food samples. Biosensors have shown many advantages over culture-independent and culture-based methods.

Their characteristics make them a promising technique in both the food industry and medical diagnostics. However, more research in biosensing techniques is still required for more biosensing performance enhancement and detection processes simplification to improve accessibility and robustness.

## Figures and Tables

**Table 1:** Current techniques used for bacterial detection in foodstuffs.

Techniques	Examples of the Techniques	Ref.
Culture-based methods	Agar plate, antibiotic susceptibility testing, blood cultures, enrichment, biochemical tests, etc.	[10]
Molecular-based assays	Loop-mediated isothermal amplification (LAMP), polymerase chain reaction (PCR), whole genome sequencing, nucleic acid sequence-based amplification (NASBA), DNA microarrays, and recombinase polymerase amplification (RPA).	[11]
Immunological-based assays	Immunochromatography assay, latex agglutination method, enzyme-linked immunosorbent assay (ELISA), and enzyme-linked fluorescent assay (ELFA).	[12]
Spectroscopy-based methods	Optical phenotyping with light diffraction technology, Raman spectroscopy, hyperspectral imaging (HIS), and near-infrared (NIR) spectroscopy.	[13]
Mass spectrometry-based methods	Liquid chromatography–mass spectrometry and matrix-assisted laser desorption ionization–time-of-flight mass spectrometry (MALDI–TOF MS).	[8]

**Table 2:** Thirty *L. mono* presumptive colonies collected in samples at day 0, day 4, and day 8 of the salad shelf-life assessment study.

Salad Code	Incubation Temperature	Number of *L. mono* Presumptive Colonies Obtained at Day 0, Day 4, and Day 8		
		Day 0	Day 4	Day 8
1	4 °C	1	0	1
2	4 °C	0	0	2
3	4 °C	2	4	0
4	12 °C	0	0	2
5	12 °C	1	1	2
6	12 °C	1	0	0
7	16 °C	0	2	3
8	16 °C	0	2	2
9	16 °C	0	2	2

**Table 3:** Results (LODs) obtained for *S. enterica*, *L. mono*, and *S. aureus* detections in broth and chicken meat samples using simplex PCR.

Species Detected	Food Sample	Limit of Detection	Detection Method	Ref.
*S. enterica*, *L. mono*, *S. aureus*	Broth	Sal: 7.3 × 10^1^ CFU/mL Lm: 6.7 × 10^2^ CFU/mL Sta: 6.9 × 10^2^ CFU/mL	Simplex PCR	[11]
*S. enterica*, *L. mono*, *S. aureus*	Chicken meat samples	Sal: 7.3 × 10^4^ CFU/mL Lm: 6.7 × 10^3^ CFU/mL Sta: 6.9 × 10^2^ CFU/mL	Simplex PCR	

**Table 4:** Results obtained for *Salmonella* detection in food samples at various concentrations.

Sample Name	*Salmonella* Concentrations	*Salmonella* Counts Detected (CFUs/mL)
Eggs	2.0 × 10^3^	1885
Eggs	2.0 × 10^2^	218
Eggs	2.0 × 10^1^	21
Chicken	2.0 × 10^3^	2070
Chicken	2.0 × 10^2^	199
Chicken	2.0 × 10^1^	21
Spiked milk	2.0 × 10^3^	1975
Spiked milk	2.0 × 10^2^	203
Spiked milk	2.0 × 10^1^	20

**Table 5:** The recovery rate demonstrates the comparison between the concentration calculated from the calibration curve to the actual concentration placed on the biosensor.

Actual Concentration log10 (CFU/mL)	Buffer		Chicken Broth		Ref.
	Calculated Concentration log10 (CFU/mL)	Recovery Rate	Calculated Concentration log10 (CFU/mL)	Recovery Rate	
2	1.97	98%	1.75	88%	[82]
3	2.87	96%	2.77	92%	
4	3.39	85%	3.78	95%	
5	4.44	89%	4.80	96%	
6	4.99	83%	5.75	96%	

**Table 6:** A summary of the biosensing approaches discussed above.

Type of Electrochemical Biosensor	Foodborne Pathogen	Food Matrix	Electrode Used	Electrode Modification	LOD	Ref.
Phage-based electrochemical biosensors	*S. Typhimurium*	Skim milk and lettuce	GCE	RBP 41, carboxylated GO, AuNPs, BSA	0.298 Log_10_ CFU/mL	[70]
Phage-based electrochemical biosensors	*S. Typhimurium*	Eggs, chicken, and milk	GDE	Phage L66, AuNPs, MPA, BSA	21 CFU/mL	[83]
Antibody-based electrochemical biosensors	*L. mono*	Milk	GE	anti-*L. mono* Ab, *L. mono* cells, HRP-labelled rabbit polyclonal Ab	≈7.5 CFU/mL	[74]
Nucleic Acid-Based Electrochemical Biosensors	*S. Typhimurium*	Pork, beef, mutton, donkey, dairy, and RTE egg products	GCE	Fc-hp, AuNPs	2.08 fg·µL^−1^	[78]
Nucleic Acid-Based Electrochemical Biosensors	*L. mono*	Fish meat	CILE	AuNPs, RGO, ssDNA/p	3.17 × 10^−14^ mol/L (3S_0_/S)	[88]
Aptamer-Based Electrochemical Biosensors	*L. mono*	Lettuce and fresh-cut fruits	GCE	Si@MB, AuNPs, Apt, BSA	2.6 CFU/mL	[90]
Cell-Based Electrochemical Biosensors	*S. Typhimurium*	Raw chicken	ITO	SsDNA, MWCNTs	10^1^ CFU/mL	[80]
Nanomaterials-Enhanced Electrochemical Biosensor	*L. mono*	Chicken broth	SPE	q-CNT, P100 Phage	10 CFU/mL	[82]
Nanomaterials-Enhanced Electrochemical Biosensor	*S. Typhimurium*	Raw chicken	GCE	rGO, CNT, ssDNA	10^1^ CFU/mL	[81]

**Table 7:** Different techniques used for *Salmonella* and *L. mono* detection at different concentrations.

Target Pathogen	Technique	LOD (CFU/mL)	Time (minutes)	Specificity	Ref.
*Salmonella*	Molecular-PCR	7.3 × 10^4^	-	High	[11]
*Salmonella*	Biosensor; Phage-based	0.298	30	High	[70]
*L. mono*	Molecular-PCR	6.7 × 10^3^	-	High	[69]
*L. mono*	Molecular-ELISA	1	100	High	[12]
*L. mono*	Molecular-ELFA	1	90	High	[12]
*L. mono*	Biosensor; Phage-based	8.4	-	High	[77]
*Salmonella*	Biosensor; DNA-based	2.08 µL^−1^	-	High	[78]
*L. mono*	Spectroscopy-SERS	1 × 10^5^	30	High	[65]
*Salmonella*	Immunological-LFA	4.1 × 10^2^	-	High	[63]
*L. mono*	Biosensor-DNA-based	2.6	-	High	[90]
*L. mono*	Biosensor-DNA-based	3.17 × 10^−14^	-	High	[12]
*S.enteritidis*	Biosensor Nanomaterial enhanced	6.7 × 10^1^	-	High	[77]
*L. mono*	Biosensor; Aptamer-based	2.6 CFU/mL	90	High	[90]
*Salmonella*	Biosensor Nanomaterial enhanced	10^1^	10	High	[78]
*L. mono*	Molecular-NGS	10^7^	-	Low	[54]

## Data Availability

The raw data supporting the conclusions of this article will be made available by the authors on request.
